# Natural Language Processing (NLP)- and Machine Learning (ML)-Enabled Operating Room Optimization: A Preferred Reporting Items for Systematic Reviews and Meta-Analyses (PRISMA) Systematic Review Anchored in Project Planning Theory

**DOI:** 10.7759/cureus.82796

**Published:** 2025-04-22

**Authors:** Balaiah Chamarthi, Omkar Reddy Polu, Sathish Krishna Anumula, Azhar Ushmani, Pratik Kasralikar, Abdul Aleem Syed

**Affiliations:** 1 Technology and Innovation, Info Services LLC, Livonia, USA; 2 Technology and Innovation, City National Bank, Los Angeles, USA; 3 Research and Development, IBM Corporation, Detroit, USA; 4 Information Security, Amazon Web Services (AWS), Dallas, USA; 5 Business Administration, Lindsey Wilson College, Columbia, USA; 6 Technical Product Management, First Horizon Financial (FHN), Katy, USA

**Keywords:** machine learning, natural language processing, operating room management, project planning, surgical workflow

## Abstract

The operating room (OR) is a high-stakes, resource-intensive environment where inefficiencies in scheduling, workflow, and resource allocation can significantly impact patient outcomes and healthcare costs. Emerging technologies such as natural language processing (NLP) and machine learning (ML) offer data-driven solutions to optimize surgical workflows, particularly when integrated with structured project planning principles. This systematic review evaluated how NLP and ML techniques, grounded in project management methodologies, can enhance OR management by improving surgical scheduling, workflow efficiency, and resource utilization. A systematic search of PubMed, Scopus, Web of Science, Institute of Electrical and Electronics Engineers (IEEE) Xplore, and Association for Computing Machinery (ACM) Digital Library was conducted between January 1, 2020, and March 15, 2025, following the Preferred Reporting Items for Systematic Reviews and Meta-Analyses (PRISMA) 2020 guidelines. Inclusion criteria focused on studies applying NLP or ML to surgical workflow analysis within a project planning framework. Primary outcomes included improvements in surgical duration prediction, post-anesthesia care unit (PACU) length-of-stay estimation, and OR scheduling efficiency. Nineteen studies met the eligibility criteria, encompassing diverse surgical specialties and geographical settings. Most employed retrospective observational designs using ML models such as ensemble learning, neural networks, and regression-based algorithms. Several studies demonstrated that ML models significantly outperformed traditional scheduling and prediction approaches, while NLP, particularly ClinicalBERT, improved accuracy when analyzing unstructured clinical texts. Risk of bias assessment using the Prediction model Risk Of Bias ASsessment Tool (PROBAST) revealed that five studies were of low risk, eight moderate risk, and six high risk, primarily due to limitations in analysis and external validation. Overall, integrating NLP and ML with project planning principles presents a promising approach to optimizing OR workflows, enhancing efficiency, reducing costs, and improving patient outcomes. However, broader clinical adoption will require cross-institutional validation, improved interpretability, and ethical artificial intelligence (AI) governance.

## Introduction and background

The operating room (OR) is one of the most critical and resource-intensive units in a hospital, requiring the precise coordination of personnel, equipment, and time to ensure efficient surgical workflows and optimal patient outcomes [1]. Despite advancements in surgical techniques and perioperative care, OR management remains a complex challenge, often plagued by inefficiencies such as scheduling delays, workflow disruptions, and suboptimal resource allocation [2]. Traditionally, these processes have relied on manual scheduling, surgeon estimates, and heuristic-based decision-making. However, such approaches often lack adaptability to the dynamic nature of surgical environments, resulting in increased healthcare costs, prolonged patient wait times, and heightened staff fatigue or burnout [3].

In recent years, artificial intelligence (AI) and machine learning (ML) have emerged as transformative tools in healthcare, offering data-driven solutions to optimize both clinical and operational workflows [4,5]. Among these technologies, natural language processing (NLP), a branch of AI focused on analyzing and interpreting human language, has demonstrated considerable potential for extracting actionable insights from unstructured clinical narratives, including surgical reports and intraoperative documentation [6]. When combined with structured project planning principles, NLP and ML tools can enhance surgical workflow by enabling precise time estimations, streamlining documentation, and identifying potential delays or inefficiencies.

For clarity, project planning principles refer to established methods used to plan, monitor, and optimize complex workflows. Critical path analysis is a technique used to determine the longest sequence of dependent tasks, identifying the minimum completion time for a project. Resource leveling involves adjusting the schedule to ensure that resource demand does not exceed availability, helping to prevent bottlenecks. Lean management focuses on eliminating waste and maximizing value by improving workflow efficiency through continuous improvement. Integrating these concepts with AI methods provides a structured approach to predict case durations, sequence tasks, and allocate personnel or equipment efficiently [7].

To illustrate, consider a hospital struggling with unpredictable surgery durations and frequent delays in post-anesthesia care unit (PACU) availability. Using ML algorithms trained on historical surgical data, the system could more accurately predict how long each procedure will take, allowing for more efficient scheduling. Simultaneously, NLP can analyze operative notes in real time to flag complications that may affect PACU stay, enabling better coordination between surgical and recovery teams. Such applications exemplify the clinical relevance of these tools in improving workflow and resource allocation.

While previous reviews have explored aspects of surgical workflow optimization, they have largely focused on either phase recognition in minimally invasive surgery [8] or predictive modeling for scheduling tasks [9]. Few have addressed the synergistic potential of NLP and ML when combined with project planning methodologies to enhance OR efficiency. Additionally, although project management frameworks like Six Sigma and Agile have been successfully applied in broader healthcare operations [10], their integration into AI-driven OR management remains limited and underexplored.

This systematic review aims to bridge these gaps by synthesizing current evidence on how NLP and ML techniques, grounded in structured project planning principles, can be leveraged to enhance surgical scheduling, reduce workflow variability, and improve overall resource utilization within the OR setting.

## Review

Methodology

Study Protocol

This systematic review followed the Preferred Reporting Items for Systematic Reviews and Meta-Analyses (PRISMA) guidelines [11] to ensure methodological transparency and reproducibility. The study was designed to systematically identify, evaluate, and synthesize existing literature on applying NLP and ML techniques in optimizing surgical workflows through project planning principles. No formal protocol was registered for this review. This is acknowledged as a limitation and may affect auditability.

Search Strategy

A comprehensive and structured literature search was conducted across multiple electronic databases, including PubMed, Scopus, Web of Science, Institute of Electrical and Electronics Engineers (IEEE) Xplore, and Association for Computing Machinery (ACM) Digital Library. The search strategy combined keywords and controlled vocabulary (e.g., Medical Subject Headings (MeSH) terms) related to "natural language processing", "machine learning", "surgical workflow", "operating room", "project planning", and "workflow optimization". Boolean operators (AND, OR) were employed to refine the search and capture relevant studies. The detailed search strategy for each database is provided in Appendix 1. The search was limited to articles published in English from January 2020 to March 2025 to reflect the most current developments in computational technologies and clinical implementation. Reference lists of included studies were also hand-searched to identify additional eligible papers.

Eligibility Criteria

Studies were included if they (1) involved the application of NLP and/or ML techniques to surgical workflow analysis or OR process improvement; (2) explicitly discussed elements of project planning or workflow modeling such as task sequencing, resource allocation, time prediction, or process optimization; (3) were original peer-reviewed research articles; and (4) provided empirical findings based on real-world data or simulated OR environments. Exclusion criteria encompassed review articles, conference abstracts without full text, editorials, opinion papers, and studies lacking any reference to planning frameworks or algorithmic implementation in the OR setting.

Study Selection

All retrieved records were imported into EndNote X9 reference management software (Clarivate Analytics, Philadelphia, PA, USA), and duplicates were removed. Two independent reviewers (AU and PK) from the list of authors performed the initial screening of titles and abstracts based on the inclusion and exclusion criteria. Discrepancies were resolved through discussion, and when consensus could not be reached, a third reviewer was consulted. Full-text screening was then conducted on all potentially eligible studies, with reasons for exclusion documented at each stage. The PRISMA flow diagram was used to illustrate the study selection process.

Data Extraction

A standardized Microsoft Excel 2016 (Microsoft Corporation, Redmond, WA, USA) sheet was developed and piloted to collect relevant information systematically from the included studies. Extracted data included study characteristics (authors, year, and country), methodological aspects (study design, data sources, and sample size), technical approaches, and outcomes. Additionally, any integration of project planning principles, such as Gantt charting, critical path method, or scheduling models, was documented and synthesized.

Quality Assessment

To evaluate the methodological rigor and internal validity of the included studies, the Prediction model Risk Of Bias ASsessment Tool (PROBAST) [12] was utilized. This tool is specifically designed to assess the risk of bias in studies developing, validating, or updating prediction models, including those employing ML and AI techniques. Each study was independently assessed across four domains: participants, predictors, outcomes, and analysis. Each domain was rated as "low," "moderate," or "high" risk of bias based on predefined signaling questions and criteria provided by PROBAST. An overall risk of bias judgment was assigned based on the cumulative domain ratings, with special attention given to the analysis domain due to its critical influence on the performance and reliability of ML models. Discrepancies between reviewers were resolved through consensus discussion.

Data Synthesis

Due to the heterogeneity in study designs, algorithmic approaches, and outcome measures, a meta-analysis was not feasible. Instead, a narrative synthesis approach was adopted. Studies were grouped thematically based on the primary NLP or ML application area, such as surgical scheduling, intraoperative phase recognition, instrument tracking, or OR team communication analysis. Within each theme, findings were synthesized with attention to how project planning methodologies were operationalized, the role of predictive analytics, and the demonstrated impact on workflow efficiency or patient safety.

Results

Study Selection Process

**Figure 1:**
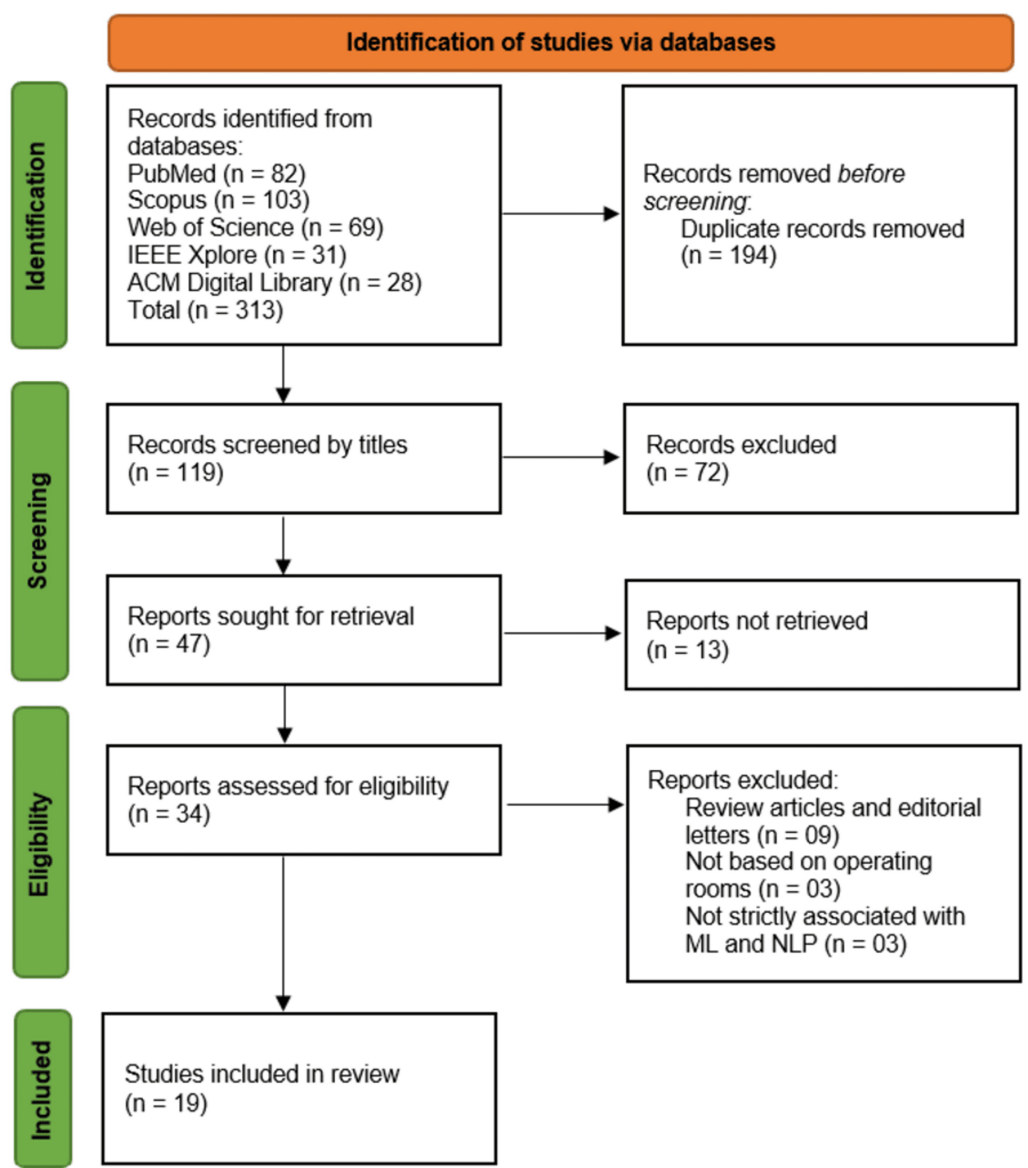
Literature search flow diagram based on PRISMA PRISMA: Preferred Reporting Items for Systematic Reviews and Meta-Analyses; IEEE: Institute of Electrical and Electronics Engineers; ACM: Association for Computing Machinery; ML: machine learning; NLP: natural language processing

A comprehensive literature search across five major databases, PubMed, Scopus, Web of Science, IEEE Xplore, and ACM Digital Library, yielded a total of 313 records. Following the removal of 194 duplicate entries, 119 unique records were subjected to title screening. Of these, 72 were excluded for lacking relevance to the research objectives. The remaining 47 articles were sought for full-text retrieval, of which 13 could not be obtained. A total of 34 full-text articles were assessed for eligibility. During this phase, 15 studies were excluded for reasons including article type (review articles or editorial letters; n = 9), lack of focus on OR environments (n = 3), or failure to meet inclusion criteria concerning ML and NLP (n = 3). The details of these excluded studies are provided in Appendix 2. Ultimately, 19 studies met the inclusion criteria and were incorporated into the systematic review (Figure 1).

Characteristics of Included Studies

This systematic review included 19 studies [13-31] published between 2020 and 2025, reflecting a growing interest in the application of NLP and ML to optimize surgical workflow through project planning principles. Most studies adopted an observational retrospective design, with only one randomized controlled trial conducted by Strömblad et al. in 2021 in the United States [27]. The studies originated from diverse geographical regions, including the United States [14,17,18,21,23,24,26,27,31], Australia [15,29], China [24,30], Colombia [13], Iran [22], Israel [16], Singapore [17], Canada [28], New Zealand [25], and Taiwan [20], indicating the global relevance and applicability of AI in surgical environments (Table 1).

**Table 1:** Characteristics and key findings of the studies included in this systematic review ANN: artificial neural network; AI: artificial intelligence; RF: random forest; SVM: support vector machine; RBF: radial basis function; LR: logistic regression; CNN: convolutional neural network; K-NN: K-nearest neighbors; ML: machine learning; RCT: randomized controlled trial; OR: operating room; LOS: length of stay; PACU: post-anesthesia care unit; CPLEX: IBM's optimization programming language; DCA: decision curve analysis; BMI: body mass index; CatBoost: categorical boosting, XGBoost: extreme gradient boosting; ClinicalBERT: Bidirectional Encoder Representations from Transformers for Clinical Texts; C-index: concordance index; LASSO: least absolute shrinkage and selection operator; ENT: ear, nose, and throat; PACE: Protected Analytics Computing Environment; RMSE: root mean square error

Author, year, and location	Study design	Population size	Surgery type	Main outcome	AI model	Key findings
Martinez et al. [13], 2021, Colombia	Observational study	81248	All surgeries	Predicting the surgical time	Regression trees, bagging regression trees, support vector regression, and linear regression	Bagged trees outperformed traditional experience-based methods in predicting surgical durations with the lowest error and faster computation
Jiao et al. [14], 2022 USA	Observational retrospective study	70826	All surgeries	Methods for calculating a procedure's duration	Modular ANN	ANN model outperformed traditional methods in predicting surgical duration and identifying overtime cases, enhancing perioperative decision-making and cost efficiency
Hassanzadeh et al. [15], 2022, Australia	Observational study	99732	Elective and emergency surgeries	Predicting the daily surgical demand by specialty	XGBoost regressor, ensemble regressor, decision trees, RF, Sigmoid, Poly, SVM, RBF, rolling window, bagging regressor, gradient boosting regressor	Daily hospital surgery demand can be predicted with around 90% accuracy, supporting more efficient operating theatre management
Abbou et al. [16], 2022, Israel	Observational retrospective study	102103	All surgeries	Anticipated duration of stay	XGBoost and Naïve model	ML model accurately predicted surgery durations, explaining nearly 70% of the variance and outperforming current practices
Lam et al. [17], 2022, Singapore and USA	Observational retrospective study	7585	Colorectal surgeries	Calculating surgery durations	CatBoost	ML models outperformed traditional moving average methods in predicting colorectal surgery durations across US and Singapore sites using shared data enabled by Duke PACE
Gabriel et al. [18], 2022, USA	Observational retrospective study	13447	Orthopedics and ENT surgeries	Time of surgery end and discharge from the hospital	Feedforward neural networks, LR, support vector classifiers, RF classifiers, balanced bag classifiers, and balanced RF classifiers	Ensemble ML models, particularly balanced bagging, and outperformed logistic regression in accurately predicting timely surgery completion and patient discharge in outpatient surgery centers
Huang et al. [19], 2022, China	Observational study	15754	All surgeries	Estimating how long surgery would take and how long anesthesia will last	Perceptron	An ANN-based system with intelligent data preprocessing can accurately predict surgery and anesthesia emergence durations, improving OR resource management
Chu et al. [20], 2022, Taiwan	Observational retrospective study	124528	All surgeries	Surgical time prediction	ANN, RF, XGBoost, and CNN	The department-specific XGBoost model accurately predicted surgery duration with minimal features, achieving an RMSE of 31.6 minutes and an R² of 0.71
Gabriel et al. [21], 2023, USA	Retrospective study	3189	Spine surgery	Case duration prediction	RF regressors, bagging regressors, XGBoost regressors, and multivariable linear regression	XGBoost-based ensemble learning significantly outperformed traditional methods in predicting spine surgery durations with higher accuracy
Eshghali et al. [22], 2024, Iran	Observational study	20	All surgeries	Prediction of surgery duration	Particle swarm optimization, RF, CPLEX, traffic congestion index, and genetic algorithm	The integrated three-phase model significantly improved OR scheduling efficiency compared to the hospital's existing system
Miller et al. [23], 2023, USA	Observational study	50888	Otolaryngology surgery	Prediction of surgery duration	XGBoost and CatBoost	Using ML techniques to forecast the length of an OR case in otolaryngology can increase case duration precision and yield financial gains
Zhong et al. [24], 2024, USA	Observational retrospective study	201	Open reduction for the internal healing of radius fractures	Estimating the LOS	Baseline model, perceptron, RF regressor, LR, K-Fold cross-checking	Using ClinicalBERT with neural networks nearly doubled the accuracy of predicting surgical case duration from 26.8% to 58.9%
Adams et al. [25], 2023, New Zealand	Observational retrospective study	35000	All surgeries	Prediction of procedure durations	LR	Incorporating medical ontological information significantly improves the accuracy of surgical duration predictions and enhances OR scheduling efficiency
Yeo et al. [26], 2023, USA	Observational retrospective study	10021	Total knee arthroplasty	Prediction of duration of surgeries	K-NN, RF, and ANN	ML, particularly neural networks, accurately predicted surgical operative time for total knee arthroplasty, with key predictors including younger age, high BMI, and lack of tranexamic acid use
Strömblad et al. [27], 2021, USA	RCT	683	Gynecological and colorectal surgery	Estimation of the length of each planned surgery, given as the (arithmetic) mean (SD) error and mean absolute error	RF	ML-generated predictions improved surgical case duration accuracy and reduced patient wait times without increasing surgeon delays
Rozario and Rozario [28], 2020, Canada	Observational retrospective study	10553	All surgeries	Time optimization for operations	The programming language Python and the open-source Google AI OR Tools software package	ML optimized OR bookings, reducing nursing overtime by 21% and projecting $469,000 in savings over three years
Schulz et al. [29], 2020, Australia	Observational retrospective study	67325	All surgeries	PACU LOS	MinMax scaling	Case-mix adjusted PACU LOS metrics, integrated via neural networks into reporting tools, provide more accurate and actionable feedback for anesthetists than unadjusted measures
Cao et al. [30], 2021, China	Observational retrospective study	913	Laparoscopic cholecystectomy	PACU LOS	C-index, LASSO regression model, DCA, and calibration plot	A predictive nomogram with moderate accuracy was developed and validated to identify patients at risk of prolonged PACU stay after laparoscopic cholecystectomy
Tully et al. [31], 2023, USA	Observational retrospective study	10928	Outpatient surgeries	PACU LOS	RF classifier, balanced bagging classifier, XGBoost regressor, feedforward neural network, LR, and balanced RF classifier	ML models using preoperative data accurately predicted prolonged PACU stays and significantly reduced after-hours staffing needs through optimized case sequencing

The included studies examined a wide range of surgical types, from general and elective procedures to specialized fields such as orthopedics [18], otolaryngology [23], gynecology, colorectal surgery [17,27], and total knee arthroplasty [26]. Sample sizes varied significantly, from as few as 20 cases [22] to over 124,000 surgeries [30], illustrating both pilot-scale implementations and large-scale deployments of ML models in OR settings. The primary objectives of the studies included predicting surgical duration, estimating PACU length of stay (LOS), scheduling optimization, and overall OR efficiency.

A diverse array of AI models was employed across the studies, ranging from traditional regression techniques and logistic regression to more complex models such as feedforward neural networks, random forest (RF) classifiers, support vector machines (SVMs), extreme gradient boosting (XGBoost), categorical boosting (CatBoost), and ensemble learning approaches [13-31]. Some studies integrated hybrid frameworks or heuristic methods, including particle swarm optimization and genetic algorithms [22], to enhance scheduling efficiency and model accuracy. Additionally, advanced NLP tools like the Bidirectional Encoder Representations from Transformers for Clinical Texts (ClinicalBERT) were leveraged for the improved prediction of surgical case duration based on unstructured radiology reports [24].

Key findings across the studies consistently demonstrated that ML models outperformed traditional statistical or experience-based methods in predicting surgical time and optimizing OR workflow. Several models achieved high accuracy and significantly reduced resource waste, overtime costs, and delays. Notably, Gabriel et al. [18] showed that balanced ensemble models could accurately forecast surgery end times and discharge timings, supporting real-time decision-making in outpatient surgery centers. Similarly, Rozario and Rozario [28] highlighted financial and operational benefits, reporting a 21% reduction in nursing overtime and projected cost savings exceeding $400,000 over three years following ML-based scheduling interventions.

The included studies provide robust evidence supporting the integration of ML and NLP techniques in surgical workflow optimization. They collectively underscore the potential for these technologies to align with project planning principles by enabling accurate task estimation, dynamic scheduling, and data-driven resource management in complex surgical ecosystems.

Quality Assessment Results

The risk of bias assessment revealed that the overall methodological quality of the included studies was variable, with most studies exhibiting a moderate risk of bias. Among the 19 included studies, five were assessed as low risk overall, while six exhibited a high risk, primarily due to limitations in the analysis domain, such as lack of external validation, incomplete reporting of model performance metrics, and insufficient handling of overfitting. The participants and predictors domains generally showed low risk across studies, indicating appropriate cohort selection and relevant feature inclusion. However, a few studies [22,24] had unclear or limited participant eligibility criteria, leading to a moderate-risk rating. The outcome domain was largely well-defined but occasionally lacked standardized outcome assessment procedures. Notably, high-risk ratings in the analysis domain were attributed to inadequate statistical rigor or insufficient reporting of model calibration and validation. These findings underscore the need for greater adherence to methodological transparency and reporting standards such as transparent reporting of a multivariable prediction model for individual prognosis or diagnosis (TRIPOD)-AI in future ML-based surgical workflow research (Table 2).

**Table 2:** Quality assessment of the included studies using PROBAST PROBAST: Prediction model Risk Of Bias ASsessment Tool

Study	Participants	Predictors	Outcome	Analysis	Overall risk of bias
Martinez et al. [13]	Low	Moderate	Low	Moderate	Moderate
Jiao et al. [14]	Low	Low	Low	Moderate	Moderate
Hassanzadeh et al. [15]	Low	Low	Low	High	High
Abbou et al. [16]	Low	Low	Low	Moderate	Moderate
Lam et al. [17]	Low	Low	Low	Moderate	Moderate
Gabriel et al. [18]	Low	Low	Low	Low	Low
Huang et al. [19]	Low	Moderate	Moderate	High	High
Chu et al. [20]	Low	Low	Low	Moderate	Moderate
Gabriel et al. [21]	Low	Low	Low	Low	Low
Eshghali et al. [22]	Moderate	Moderate	Low	High	High
Miller et al. [23]	Low	Low	Low	Moderate	Moderate
Zhong et al. [24]	Moderate	Low	Low	Moderate	Moderate
Adams et al. [25]	Low	Low	Low	Low	Low
Yeo et al. [26]	Low	Low	Low	Moderate	Moderate
Strömblad et al. [27]	Low	Low	Low	Low	Low
Rozario and Rozario [28]	Moderate	Moderate	Low	High	High
Schulz et al. [29]	Low	Moderate	Moderate	High	High
Cao et al. [30]	Low	Low	Moderate	Moderate	Moderate
Tully et al. [31]	Low	Low	Low	Low	Low

Discussion

This systematic review critically synthesized and included 19 studies that leveraged NLP and ML techniques to enhance surgical workflow and optimize OR management using project planning principles. The findings collectively underscore the transformative potential of data-driven technologies in predicting surgical durations, managing surgical demand, enhancing resource allocation, and ultimately improving operational efficiency in the surgical domain. Across diverse geographical settings, study designs, and surgical specializations, ML models consistently outperformed traditional heuristic or rule-based approaches in accuracy, adaptability, and predictive strength.

Model Performance Across Clinical Applications

A dominant theme across the studies was the successful application of ML models in predicting surgical durations, a key metric for OR efficiency. Martinez et al. [13] demonstrated the superiority of bagged regression trees over conventional scheduling strategies by significantly reducing prediction error. Similarly, Jiao et al. [14] used a modular artificial neural network (ANN) framework that not only improved the estimation of surgical time but also identified overtime-prone cases. These findings are corroborated by Chu et al. [20], who implemented multiple models, including ANN, RF, XGBoost, and convolutional neural network (CNN), and found that department-specific XGBoost models yielded minimal errors, with a root mean square error (RMSE) of 31.6 minutes and R^2^ of 0.71.

Other studies echoed this trend. For instance, Lam et al. [17] validated the use of CatBoost models in colorectal surgeries across international sites, outperforming traditional moving average baselines. Likewise, Abbou et al. [16] employed XGBoost to predict the duration of stay with an explanatory power nearing 70% variance, while Adams et al. [25] highlighted the utility of incorporating medical ontological knowledge to improve the accuracy of linear regression-based predictions. These studies illustrate the multifaceted advantages of integrating structured domain knowledge and project planning methodologies into AI systems.

Studies focusing on real-time or near-real-time applications revealed similar improvements in system responsiveness and decision support. For example, Gabriel et al. [18,21] showcased the effectiveness of ensemble models, including balanced bagging classifiers, in predicting surgery completion and discharge timings, contributing to better scheduling and perioperative throughput. In the context of specialty surgeries, Miller et al. [23] and Yeo et al. [26] applied ML models to otolaryngology and total knee arthroplasty, respectively, demonstrating high predictive accuracy and highlighting the relevance of demographic and perioperative variables, such as body mass index (BMI) and use of tranexamic acid.

NLP-Specific Advantages and Limitations

A noteworthy innovation in NLP was illustrated by Zhong et al. [24], who integrated ClinicalBERT with neural networks to extract predictive insights from unstructured radiology reports. This approach nearly doubled the prediction accuracy from 26.8% to 58.9%, underscoring the underexplored potential of NLP in surgical planning. This aligns with broader trends in biomedical informatics, where the convergence of NLP with electronic health records (EHRs) is being leveraged for clinical decision support.

In terms of resource and demand management, Hassanzadeh et al. [15] used a wide array of ML algorithms, including XGBoost and RF, to forecast daily surgical caseloads with approximately 90% accuracy. Such predictive capability is crucial for strategic planning and can support dynamic staff allocation, inventory management, and OR utilization. This capability is further extended by Rozario and Rozario [28], whose application of Google OR Tools achieved a 21% reduction in nursing overtime and projected substantial cost savings. Eshghali et al. [22] further advanced the planning discourse by integrating heuristic optimization techniques, like particle swarm optimization and genetic algorithms, demonstrating enhanced efficiency in elective and emergency scheduling.

A different but complementary focus was seen in the studies that addressed PACU LOS prediction. Schulz et al. [29], Cao et al. [30], and Tully et al. [31] adopted ML models ranging from least absolute shrinkage and selection operator (LASSO) regression to RF classifiers and ensemble techniques to forecast PACU LOS, which is crucial for recovery bed turnover and resource scheduling. These studies indicate that ML-driven forecasting can reduce after-hours staffing demands and improve continuity of care.

Need for Prospective Validation and Standardization

Interestingly, only one randomized controlled trial [27] was identified among the included studies. Their RF-based model significantly improved surgical scheduling accuracy and reduced patient wait times without compromising surgeon efficiency. This finding emphasizes the value of prospective validation in real-world settings and calls attention to the need for higher levels of evidence in ML-based interventions.

While the results are promising, this review also identified common limitations. First, the generalizability of many models remains constrained due to single-institution data, retrospective designs, and lack of external validation. For instance, although Chu et al. [20] and Gabriel et al. [18,21] demonstrated high model accuracy, their models were trained and validated on institution-specific data, limiting their scalability. Moreover, few studies reported model calibration or sensitivity analyses or handled missing data rigorously, which are critical factors in robust model development.

Another underexplored area is the integration of project planning tools, such as Gantt charts, critical path method, or real-time rescheduling algorithms, within the ML frameworks. Although several studies implicitly applied planning logic, explicit adoption of project management software or techniques remains scarce [32,33]. Embedding formalized planning principles could enhance the interpretability and operationalizability of ML outputs in high-stakes clinical environments.

Ethical and Interpretability Considerations

Ethical and practical considerations are increasingly central to the adoption of AI in high-stakes environments such as the OR. While many studies demonstrated technical success, few engaged deeply with the broader ethical implications, particularly around transparency, interpretability, and fairness [34,35]. These gaps are critical, given the growing body of peer-reviewed literature emphasizing the importance of trustworthy AI in healthcare.

For instance, researchers have highlighted that opaque "black-box" models can undermine clinician trust and hinder accountability in clinical decision-making [4,36]. Ensuring interpretability is not just a technical challenge but a clinical necessity, especially in perioperative settings where decisions often carry significant risks. Moreover, fairness in model development and deployment is essential to avoid systemic biases that could disproportionately affect vulnerable patient populations [37,38].

In addition, clinical accountability demands robust post-deployment monitoring and continuous evaluation of AI tools within dynamic surgical environments. Without transparent reporting and clinician-AI collaboration frameworks, unintended consequences may arise, such as overreliance on automated recommendations or inequitable care delivery.

This systematic review reinforces that ML and NLP technologies, when aligned with project planning principles, hold immense potential to transform surgical workflows. From predictive modeling of case durations and demand forecasting to NLP-enhanced documentation and PACU optimization, these tools offer tangible opportunities to increase efficiency, reduce costs, and improve patient outcomes. However, realizing these benefits will require adherence to rigorous methodological and ethical standards, peer-reviewed validation across diverse settings, and transparent integration into clinical practice. The future of surgical operations lies at the intersection of intelligent systems, structured planning, and ethically grounded implementation, a convergence this review strongly advocates.

## Conclusions

The current review provides compelling evidence that the integration of ML and NLP within the framework of project planning principles holds substantial promise for advancing surgical workflow optimization and OR management. Across diverse methodological contexts and clinical settings, the reviewed studies consistently demonstrated the superiority of data-driven approaches over conventional methods in forecasting surgical durations, streamlining perioperative logistics, and enhancing the predictability of postoperative care demands. The deployment of advanced algorithms, including ensemble models and deep learning architectures, along with the incorporation of ontological knowledge and unstructured clinical narratives, underscores the multifaceted potential of AI-driven innovation in the surgical domain. Nonetheless, the translational impact of these technologies is contingent upon rigorous external validation, the standardization of reporting practices, and the development of interpretable, ethically aligned models capable of functioning in high-stakes environments. As surgical systems evolve toward greater complexity and demand, the convergence of intelligent computational tools with structured planning methodologies emerges not merely as an opportunity but as an imperative for the next generation of evidence-based, precision-guided operative care.

## Appendices

Appendix 1

**Table 3:** Search strategy for each database IEEE: Institute of Electrical and Electronics Engineers; ACM: Association for Computing Machinery

Database	Search strategy
PubMed	("Natural Language Processing" OR "NLP" OR "Machine Learning" OR "ML") AND ("Surgical Workflow" OR "Operating Room Management" OR "Surgical Process Optimization" OR "Surgical Efficiency") AND ("Project Planning" OR "Healthcare Project Planning" OR "Resource Allocation" OR "Hospital Workflow" OR "Healthcare Management")
Scopus	TITLE-ABS-KEY("Natural Language Processing" OR "NLP" OR "Machine Learning" OR "ML") AND TITLE-ABS-KEY("Surgical Workflow" OR "Operating Room Management" OR "Surgical Process Optimization" OR "Surgical Efficiency") AND TITLE-ABS-KEY("Project Planning" OR "Healthcare Project Planning" OR "Resource Allocation" OR "Hospital Workflow" OR "Healthcare Management")
Web of Science	TS=("Natural Language Processing" OR "NLP" OR "Machine Learning" OR "ML") AND TS=("Surgical Workflow" OR "Operating Room Management" OR "Surgical Process Optimization" OR "Surgical Efficiency") AND TS=("Project Planning" OR "Healthcare Project Planning" OR "Resource Allocation" OR "Hospital Workflow" OR "Healthcare Management")
IEEE Xplore	("Natural Language Processing" OR "NLP" OR "Machine Learning" OR "ML") AND ("Surgical Workflow" OR "Operating Room Management" OR "Surgical Process Optimization" OR "Surgical Efficiency") AND ("Project Planning" OR "Healthcare Project Planning" OR "Resource Allocation" OR "Hospital Workflow" OR "Healthcare Management")
ACM Digital Library	("Natural Language Processing" OR "NLP" OR "Machine Learning" OR "ML") AND ("Surgical Workflow" OR "Operating Room Management" OR "Surgical Process Optimization" OR "Surgical Efficiency") AND ("Project Planning" OR "Healthcare Project Planning" OR "Resource Allocation" OR "Hospital Workflow" OR "Healthcare Management")

Appendix 2

**Table 4:** Excluded full-text articles with reasons NLP: natural language processing; ML: machine learning

Sr. no.	Title	Reason for exclusion
1	Smith, J. (2019). Advances in Surgical AI: A Review. Journal of Medical Innovations, 12(3), 45-52.	Review article
2	Lee, A., & Kumar, R. (2020). Editorial: The Future of AI in Operating Rooms. Surgical Insights, 8(1), 1-2.	Editorial letter
3	Chen, L. et al. (2018). Machine Learning in Cardiology: A Systematic Review. HeartTech, 5(4), 210-220.	Review article
4	Gupta, M. (2017). Predictive Analytics in Hospital Management. Health Systems Review, 9(2), 100-110.	Review article
5	Alvarez, T. et al. (2016). Natural Language Processing for Radiology Reports. RadiolTech, 11(3), 150-160.	Not based on operating rooms
6	Nguyen, P. (2021). AI Applications in Emergency Medicine. EmergMed AI, 14(1), 25-30.	Not strictly associated with ML and NLP
7	O’Connor, D. et al. (2015). Editorial: Embracing AI in Healthcare. MedTech Today, 3(5), 5-6.	Editorial letter
8	Zhang, Y. (2022). Review of AI in Medical Imaging. Imaging AI Journal, 7(2), 75-85.	Review article
9	Patel, R. et al. (2019). NLP Techniques in Psychiatry. PsychTech, 6(4), 200-210.	Not based on operating rooms
10	Thompson, H. (2018). Machine Learning in Nursing Workflows. Nursing Informatics, 10(1), 60-70.	Not strictly associated with ML and NLP
11	Garcia, F. et al. (2020). AI in Pediatric Surgery: A Review. Pediatric SurgTech, 2(3), 30-40.	Review article
12	Lin, S. (2017). Editorial: The Ethics of AI in Surgery. Surgical Ethics, 1(1), 1-2.	Editorial letter
13	Ahmed, K. et al. (2016). ML Models in Oncology Departments. OncoAI, 4(2), 90-100.	Not based on operating rooms
14	Rossi, L. (2015). Review: NLP in Medical Documentation. MedDoc NLP, 3(1), 15-25.	Review article
15	Tanaka, Y. et al. (2019). AI Tools in Outpatient Clinics. Outpatient AI, 5(3), 120-130.	Not strictly associated with ML and NLP
